# Primary Clear Cell Chondrosarcoma of the Spine: A Case Report of a Rare Entity and a Review of the Literature

**DOI:** 10.1155/2012/693137

**Published:** 2012-10-09

**Authors:** Nikolaos A. Paidakakos, Aristides Rovlias, Evaggelos Rokas, Spyridon Theodoropoulos, Patroklos Katafygiotis

**Affiliations:** ^1^Department of Neurosurgery, Asclepeion Voulas General Hospital, Vassileos Pavlou 1, Voula, 16673 Athens, Greece; ^2^Department of Pathology, Asclepeion Voulas General Hospital, Vassileos Pavlou 1, Voula, 16673 Athens, Greece

## Abstract

Chondrosarcoma is the third most common primary malignant bone tumor after osteosarcoma and Ewing's sarcoma. Clear cell chondrosarcoma is a rare subtype variant of chondrosarcoma, most commonly encountered in the proximal part of the femur or humerus. Vertebral involvement is exceedingly rare and shows a predilection for the thoracic spine. We report the case of a woman with clear cell chondrosarcoma of the thoracic spine, which has been surgically excised, and review the pertinent literature (PubMed). Although it has a reasonably benign biological behavior, clear cell chondrosarcoma needs to be treated as a malignancy. The best treatment for spinal chondrosarcoma is surgery. It should be promptly and adequately resected. Gross-total resection should be the ultimate surgical goal. Radiation therapy should also be considered, especially in the case of subtotal resection or inoperable lesions. In conclusion, it is important to keep in mind this entity in the differential diagnosis of spinal tumors, in order to optimize treatment planning. With adequate treatment, local recurrence rates as low as 20% can be achieved.

## 1. Introduction

Clear cell chondrosarcoma is a rare subtype variant of chondrosarcoma, most commonly encountered in the proximal part of the femur or humerus. Vertebral involvement is exceedingly rare. Surgical treatment is of paramount importance, since it is considered as a low-grade malignancy with high survival rates but may be challenging due to its proximity to the spinal cord and nerve roots. We report the case of a woman with clear cell chondrosarcoma of the thoracic spine and review the pertinent literature.

## 2. Case Presentation

A female patient, 61 years old, presented to our emergency department complaining of aggravating weakness of the lower limbs and back pain of one month duration. Upon admittance she was unable to stand and walk. She had visited a physician twice in this period, but she had only been administered with NSAIDs and analgesics. A careful physical evaluation revealed muscle weakness and hypoesthesia in both lower limbs and a 5th thoracic sensory level.

**Figure 1:**
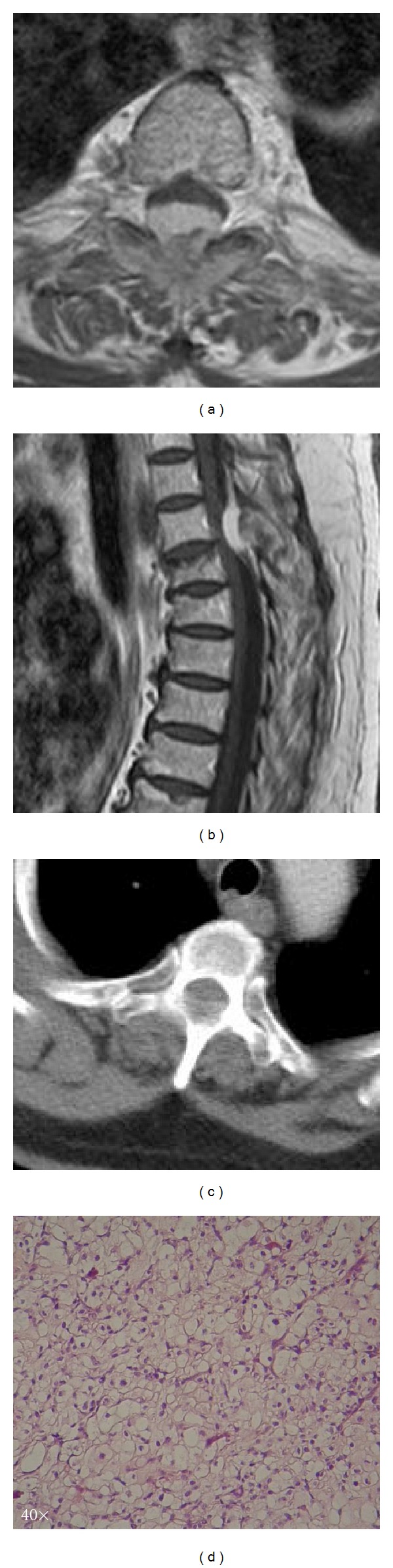
(a) Axial T2 weighted MR image of the lesion at Th3 level, demonstrating significant spinal cord compression; (b) Sagittal contrast enhanced T1 weighted MR image of the lesion, showing spinal cord compression at Th3-Th4 level; (c) Axial CT scan of the lesion at Th3 level, demonstrating spinal canal involvement, bony erosion, and paravertebral extension of the tumor; (d) H/E ×40 Clear cell chondrosarcoma characterised by tumor cells with variably clear cytoplasm.

Plain radiographs of the thoracic spine did not show anything other than a cuneiform degeneration of the 4th thoracic vertebra. A computed tomography scan revealed a spinal mass, with osteolytic erosion of the posterior elements of the 3rd and 4th thoracic vertebrae, and paravertebral extension (Figure 1(c)). Magnetic resonance sequences unveiled a contrast-enhanced extradural mass, with significant spinal cord compression at the aforementioned levels (Figures 1(a) and 1(b)). The lesion presented radiological features of metastatic origin. Nonetheless, thorough examinations (tumor markers, chest/abdomen CT, scintigraphy) were negative for presence of primary disease.

The patient underwent 3rd and 4th thoracic laminectomy, with consecutive gross-total excision of the mass with the aid of the operating microscope. Infiltrated surrounding tissue was also surgically debrided until margins free of tumor were obtained. Pathology was compatible with clear cell chondrosarcoma, mixed with conventional grade I chondrosarcoma (Figure 1(d)).

**Figure 2:**
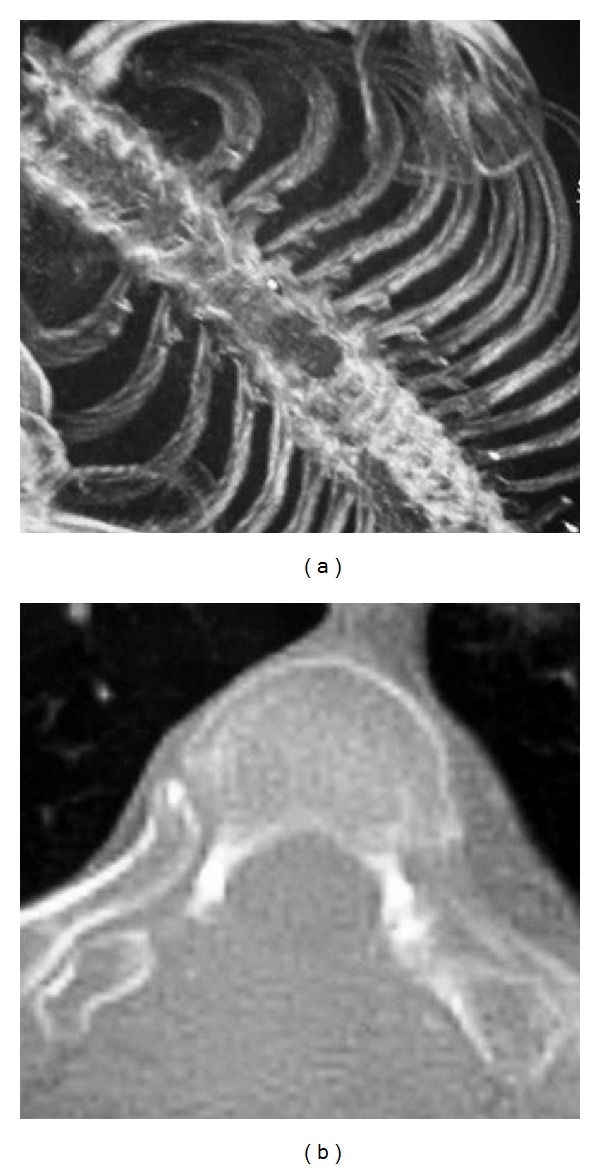
Postoperative (1 year) CT scan of the lesion at Th3-Th4 level, demonstrating laminectomy and absence of local recurrence.

She showed immediate relief of her symptoms, being able to walk on the second postoperative day. A mild paresis of the left lower limb persisted in the immediate postoperative period. The oncological evaluation suggested adjuvant radiotherapy to minimize the risk of potential residual tumor. Hence, the patient was referred for and received postoperative three-dimensional conformal radiation therapy (50 Gy) two months after surgery. At one year from the operation, the patient is doing physically well and is free of local recurrence (Figure 2) or secondary disease. 

## 3. Discussion 

Chondrosarcoma is the third most common primary malignant bone tumor after osteosarcoma and Ewing's sarcoma. However, the incidence of spinal chondrosarcomas is estimated to be from 2% to 12% in various series [1]. It can occur within all regions of the spine, but the thoracic spine is the most frequent localization, followed by the cervical and lumbar region [2]. Within the vertebra, lesions may arise in the vertebral body (5%), the posterior elements (40%), or both (45%), since there are three growth centers in each vertebra from which the tumor originates [1]. Chondrosarcoma located in the posterior elements typically arises from an underlying benign chondral lesion.

Clear cell chondrosarcoma is a rare subtype of chondrosarcoma first described by Unni et al. in 1976 [3]. It accounts for 2 to 5.4% of all chondrosarcomas [4]. Men are affected more than women, and it most commonly occurs during the third and fourth decades of life [5]. Clear cell chondrosarcoma is most commonly localized in the epiphyseal region of long bones, particularly femur and humerus. Presentation as a primary tumor of the osseous spine is rare, with only a few cases reported in the literature [4–11].

Histologically clear cell chondrosarcoma is characterized by benign giant cells alongside tumor cells with clear or granular cytoplasm, mixed with conventional chondrosarcoma in 50% of the cases. The cells present with rounded and centrally located nuclei. In cases in which the spine is involved, bone formation and cartilaginous components are minimal or even absent [3]. Vascularity is a common feature in this tumor [1]. On the molecular level, recent studies have shown that there is evidence of extra copies of chromosome 20 and loss or rearrangements of 9p. Also, expression of PTHLH, PDGFIHH, Runt-related transcription factor 2, and matrix metalloproteinase 2 was found [9, 12].

The most common presenting symptom in chondrosarcoma is pain in the area of the lesion. The pain is often insidious in nature and can be present for weeks to years. Other complaints include a palpable mass and neurologic deficit in half of the patients [2]. The neurologic presentation can range from radicular pain to frank weakness.

Although clear cell chondrosarcomas are considered low-grade malignant tumors, dedifferentiation into a more aggressive neoplasm can occur. Distant metastases have been reported [13]. Even though metastases are rare, they may occur up to 20 years following initial diagnosis; consequently, long-term followup is required [14]. Furthermore, having a reasonably benign biological behavior, clear cell chondrosarcoma needs to be treated as a malignancy. The best treatment for spinal chondrosarcoma is surgery. En bloc resection is associated with longer survival rates [15]. Local recurrence rates as low as 20% can be achieved. Unfortunately en block resection is not always feasible, due to limitations of location, compromise of stability, and risk of neurological deficit [16]. Thus, in some cases intralesional curettage is more appropriate. In this regard, a set of selection criteria have been proposed by Boriani et al. [15]. They include circumferential spinal canal involvement, the potential of cord ischemia due to ligation of the segmental artery, and the need for spinal cord ligation to complete en block resection. Unfortunately, results following curettage are poor. 

Chondrosarcomas are poorly responsive to chemotherapy and radiation [17]. However, high-dose proton and photon radiation therapy may play a role in local control of chondrosarcomas, especially in the case of subtotal resection or inoperable lesions [18].

Finally, appropriate staging is essential for diagnosis and treatment and includes radiologic and histological information. The appropriate imaging studies allow for better understanding of the extent of the lesion as well as differential diagnosis, which includes metastatic lesions, angioblastic meningioma, osteosarcoma, Ewing's sarcoma, and hemangiopericytoma [1]. Imaging includes plain radiographs, CT, and MRI to evaluate the tumor. Additional imaging of the chest, abdomen, pelvis, and rest of spinal cord, including scintigraphy, is also necessary to exclude secondary disease. When a histological diagnosis is needed preoperatively to aid in surgical planning, image-guided needle biopsy is preferred to excisional biopsy, since the latter can adversely alter prognosis and survival [15].

## 4. Conclusion

Primary clear cell chondrosarcoma of the spine is a rare encounter in clinical practice; being a rather low-grade malignancy, it benefits greatly from prompt and adequate treatment. Thus, it is important to keep in mind this entity in the differential diagnosis of spinal tumors, in order to optimize treatment planning. Clear cell chondrosarcoma is a surgical disease. Gross-total resection should be the ultimate surgical goal, although not always is it feasible due to the nobility of nearby structures. Adjuvant radiotherapy should also be taken into consideration in the management of this tumor.

## Abbreviations

CT: Computed tomography
MR: Magnetic resonance
H/E: Hematoxylin/eosin
NSAIDs: Non steroidal anti-inflammatory drugs
Th: Thoracic
Gy: Gray
PTHLH: Parathyroid hormone-like hormone
PDGFIHH: Platelet-derived growth factor/Indian hedgehog.
